# Eyesight quality and Computer Vision Syndrome

**DOI:** 10.22336/rjo.2017.21

**Published:** 2017

**Authors:** Camelia Margareta Bogdănici, Diana Elena Săndulache, Corina Andreea Nechita

**Affiliations:** * Surgery II Department, Discipline of Ophthalmology, “Grigore T. Popa” University of Medicine and Pharmacy, Iași, Romania; “Sf. Spiridon” Hospital, Iaşi, Romania

**Keywords:** eyesight quality, refractive errors, ocular congestion, gadgets, Computer Vision Syndrome

## Abstract

The aim of the study was to analyze the effects that gadgets have on eyesight quality. A prospective observational study was conducted from January to July 2016, on 60 people who were divided into two groups: Group 1 – 30 middle school pupils with a mean age of 11.9 ± 1.86 and Group 2 – 30 patients evaluated in the Ophthalmology Clinic, “Sf. Spiridon” Hospital, Iași, with a mean age of 21.36 ± 7.16 years. The clinical parameters observed were the following: visual acuity (VA), objective refraction, binocular vision (BV), fusional amplitude (FA), Schirmer’s test. A questionnaire was also distributed, which contained 8 questions that highlighted the gadget’s impact on the eyesight. The use of different gadgets, such as computer, laptops, mobile phones or other displays become part of our everyday life and people experience a variety of ocular symptoms or vision problems related to these. Computer Vision Syndrome (CVS) represents a group of visual and extraocular symptoms associated with sustained use of visual display terminals. Headache, blurred vision, and ocular congestion are the most frequent manifestations determined by the long time use of gadgets. Mobile phones and laptops are the most frequently used gadgets. People who use gadgets for a long time have a sustained effort for accommodation. A small amount of refractive errors (especially myopic shift) was objectively recorded by various studies on near work. Dry eye syndrome could also be identified, and an improvement of visual comfort could be observed after the instillation of artificial tears drops. Computer Vision Syndrome is still under-diagnosed, and people should be made aware of the bad effects the prolonged use of gadgets has on eyesight.

## Introduction

**Fig. 1:**
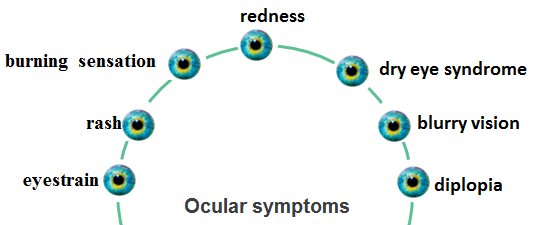
Ocular symptoms

It is estimated that 60 million people were diagnosed with Computer Vision Syndrome (CVS) [**1**]. CVS represents a group of visual (**Fig. 1**) and extraocular symptoms associated with the sustained use of visual display terminals [**2**]. 

**Fig. 2:**
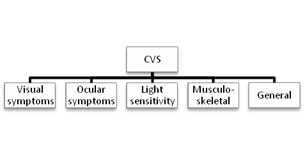
CVS symptoms

These symptoms are [**2**]: visual symptoms, ocular symptoms, asthenopia, light sensitivity, musculoskeletal and general symptoms (**Fig. 2**). The visual symptoms are related to blurred vision: constant blurred vision, post work distance blur and intermittent blurred vision at near. Ocular surface related symptoms are itching eyes, burning eyes, foreign body sensation and sore eyes. Patients who work in front of a VDT for more than 4 hours per day can develop dry eye disease. Ocular complaints can also be related with the dysfunction of meibomian glands [**3**]. Some patients complain of excessive tears and excessive blinking [**4**]. 

The prevalence of asthenopia among the gadgets users is estimated between 55% and 81%. Prolonged use of gadgets can cause removal of the near point of convergence, deviation of phoria for near vision and inaccurate accommodative response [**2**]. These modifications are only temporary and do not have a permanent effect on accommodation [**5**]. Some of the musculoskeletal symptoms are represented by neck pain, back pain and shoulder pain, which are frequently related to the use of computers [**2**]. CVS can also generate general symptoms that are not directly linked with eyes and occur towards the end of the day like irritability, increased nervousness, general fatigue, drowsiness. CVS is temporary and it is heightened by inadequate lighting condition [**4**]. Children use computers more and more for both school and recreation purposes and start to develop early symptoms similar to adults. They also develop musculoskeletal symptoms because computer stations are mostly designed for adult use and may not be suitable for children [**2**]. Many of the symptoms in CVS can be prevented with proper eye care, patient education and by providing a proper working environment [**6**].

## Material and methods

The study was a prospective observational one, conducted from January to July 2016, on 60 people, who were divided into two groups: Group 1 (screening), consisting of 30 middle school students from “Dimitrie A. Sturdza” School, Iași, and a second group (Group 2), of 30 patients from “Sf. Spiridon” Ophthalmology Clinic Hospital, Iași. Selected patients from Group 1 had a mean age of 11.9 ± 1.86 years (limits 8-15 years), and from Group 2, the mean age was 21.36 ± 7.16 years (limits 8-43 years). Students in Group 1 were all from urban areas and the majority of the patients in Group 2 (53%) were from rural areas. A questionnaire was used to collect data regarding the gadget’s impact on eyesight. We were particularly interested in the types of display used, the amount of time spent in front of the device per day and the symptoms felt by the patients. The following investigations were made in all the selected cases: visual acuity (VA) corresponding with Snellen test, objective refraction (OR), binocular vision (BV), fusional amplitude (FA) and Schirmer’s test. Refraction values were calculated by using spherical equivalent (SEq), which represented the sphere’s value plus half of the cylinder’s value. Data were statistically analyzed by using Student’s t-test (statistically significant at p ≤ 0.05).

## Results

In Group 1, the mean visual acuity (VA) of the right eye (RE) without correction was 0.91 ± 0.42 (limits between 0.16 and 1.5). For the left eye (LE), the mean VA without correction was 0.89 ± 0.44 (limits between 0.16 and 1.5). The mean VA with correction for the RE was 1.08 ± 0.20 (limits between 0.8 and 1.5) and for the LE 1.08 ± 0.22 (limits between 0.6 and 1.5). In Group 2, the mean VA without correction for the RE was 0.93 ± 0.29 (limits between 0.1 and 1.6) and for the LE was 0.88 ± 0.31 (limits between 0.05 and 1.6). VA with correction for the RE was 1.01 ± 0.13 (limits between 0.7 and 1.2) and for the LE was 0.97 ± 0.3 (limits between 0.1 and 1.5). P values were not statistically significant (**Table 1**).

**Table 1:** Comparative values of visual acuity (without and with correction)

	Without correction		With correction	
	Group 1	Group 2	Group 1	Group 2
Average VA - RE	0.91±0.42	0.93±0.29	1.08±0.20	1.01±0.13
P value Group 1 vs. Group 2 (RE)		0.813		0.380
Average VA - LE	0.89±0.44	0.88±0.31	1.08±0.22	0.97±0.3
P value Group 1 vs. Group 2 (LE)		0.935		0.337

By analyzing the VA, it was found that there were more patients with amblyopia in Group 1 than in Group 2. The following classification was used for amblyopia: relative amblyopia – VA between 0.8 and 0.9, easy amblyopia – VA between 0.5 and 0.8, medium amblyopia – VA between 0.3 and 0.5, serious amblyopia – VA between 0.1 and 0.3 and severe amblyopia - VA less than 0.1.

In Group 1, 16.66% had relative amblyopia and 6.66% medium amblyopia in the RE, and 10% had relative amblyopia, 10% easy amblyopia and 3.33% medium amblyopia in the LE. In Group 2, 6.66% had relative amblyopia and 3.33% easy amblyopia in the RE, and 6.66% had relative amblyopia and 3.33% severe amblyopia in the LE. The present study was also used as screening for diagnosing amblyopia.

Regarding the optical correction, more than 60% in both groups had an optical correction (60% in Group 1 and 67% in Group 2). In Group 1, the refractive errors (in SEq) for the RE had a mean value of 0.675 ± 0.921 D and for the LE 0.837 ± 1.075 D. In Group 2, the mean value for the RE was 0.983 ± 1.245 D and for the LE was 1.131 ± 1.287 D (**Fig. 3a**,**b**).

**Fig. 3a:**
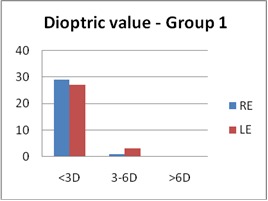
Refractive errors - Group 1 (SEq)

**Fig. 3b:**
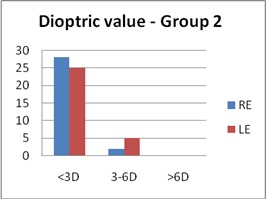
Refractive errors - Group 2 (SEq)

In both groups, the main type of ametropia was astigmatism (66.6% in Group 1 and 68.3% in Group 2). In 26.7%, myopic astigmatism was found for the LE in Group 1, and in 33.3% in Group 2. Other types of ametropia found were myopia (5% in Group 1 and 11.6 % in Group 2), hyperopia (13.3% in both groups), and anisometropia (15% in Group 1 and 6.6% in Group 2). Anisometropia could be a cause for amblyopia, which could explain the higher number of patients with amblyopia in Group 1 compared to Group 2, as it was previously seen. 

**Fig. 4:**
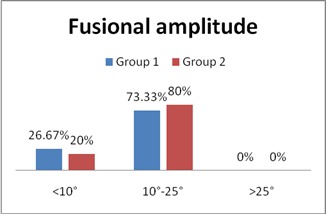
Distribution of cases according to FA

All the patients in both groups had binocular vision with all three stages except for one patient in Group 1, who only had first stage of binocular vision (**Fig. 4**). Fusional amplitude (FA) had values under 25º for all the patients in both groups. When the value of FA dropped under 30º, patients could complain of accommodative asthenopia symptoms such as eye pain, blurry vision, frontal headache (p = 0.216 was not statistically significant). 

Among subjective ocular signs, blurred vision prevailed in both groups, with a statistically significant difference (p = 0.0039). Other subjective ocular signs present were burning sensation, diplopia, and foreign body sensation.

In Group 2, 63.33% of the patients had headache, while in Group 1, 30% (p = 0.007 - statistically significant). Ocular congestion was present in 26.66% of the patients in Group 1 and 20% in Group 2. Also, in 30% of the cases, both groups presented excessive blinking. Dry eye syndrome was identified in 10% of the subjects in Group 1 and in 16% of those in Group 2 (**Fig. 5a**,**b**).

**Fig. 5a:**
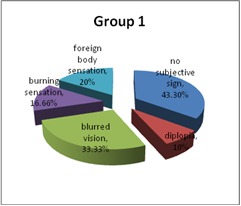
Subjective ocular signs – Group 1

**Fig. 5b:**
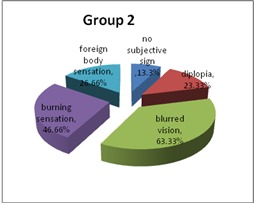
Subjective ocular signs – Group 2

**Fig. 6:**
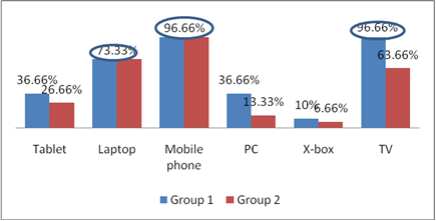
Distribution of cases according to the type of gadgets used

Mobile phones, TV, and laptops are the most frequently used gadgets (**Fig. 6**). The average time spent in front of a device per day was 2.56 h in Group 1, and 3.3 h in Group 2. In Group 2, 50% of the patients affirmed that they spend more than 4 h per day in front of a device (p = 0.004 - statistically significant). 

## Discussions

Computers are part of modern life, but many people are experiencing a variety of ocular symptoms related to computer use, such as eyestrain, tired eyes, irritation, redness, blurred vision, and double vision [**5**]. More and more people are now using different types of gadgets, especially smart phones. With the unprecedented growth of users of handheld devices, it is estimated that almost 84% of the world’s population will use these by the end of 2018 [**2**]. In our study, mobile phones, TV, and laptops were the most frequently used gadgets by our patients. The average time spent in front of a device per day was between 2.6 and 4 h.

Using gadgets for a long time can produce ocular asthenopia. In fact, visual complaints were reported by 75% of the users who work 6–9 hours in front of a screen compared to 50% of the other workers. A small, transient myopic shift seems to occur after computer use, but its significance with respect to creating permanent myopic change is unknown [**7**]. In this study, among subjective ocular signs, blurred vision prevailed in both groups, with a statistically significant difference (p = 0.0039). Other subjective ocular signs present were burning sensation, diplopia, and foreign body sensation.

In our study, myopic astigmatism for LE was found in 26.7% of the cases in Group 1, and in 33.3% in Group 2. Other types of ametropia found were myopia (5% in Group 1 and 11.6 % in Group 2). It is reported that during a prolonged deskwork on a computer or other gadgets, sustained effort for accommodation is required. A small amount of myopic shift has been objectively recorded by various studies on near work induced transient myopia, which produces ocular fatigue [**2**,**8**]

In this techno-age, children as young as two years old are given touch screen devices like iPads to play with and learn [**9**] The mean age in our selected group was 11.9 ± 1.86 years (limits 8-15 years). It is important to control the lacrimal film and how the patient blinks, because dry eye is intimately related to CVS as either cause or effect [**10**].

The major strength of this study was that it was the first prospective observational study performed in Romania that analyzed the effects that gadgets have on eyesight quality. This study was limited by the small sample size, and the fact that it did not include a follow up examination of the patients.

## Conclusions

Myopic astigmatism and myopia were the main types of ametropia in both study groups. The fusional amplitude was one of the main indicators necessary for the study. Following the use of gadgets, the subjective signs that stood out were blurred vision, headache, and ocular congestion. Mobile phones and laptops were the most frequently used gadgets with an average usage of 1 to 4 hours per day. Computer Vision Syndrome is still under-diagnosed, and people should be made aware of the bad effects the prolonged use of gadgets has on eyesight.
